# Improving Cadmium Resistance in *Escherichia coli* Through Continuous Genome Evolution

**DOI:** 10.3389/fmicb.2019.00278

**Published:** 2019-02-20

**Authors:** Weitong Qin, Jintong Zhao, Xiaoxia Yu, Xiaoqing Liu, Xiaoyu Chu, Jian Tian, Ningfeng Wu

**Affiliations:** Biotechnology Research Institute, Chinese Academy of Agricultural Sciences, Beijing, China

**Keywords:** molecular evolution, cadmium resistance, minimum inhibitory concentration, HtpX, Gor

## Abstract

Cadmium (Cd) is a heavy metal that is extremely toxic to many organisms; however, microbes are highly adaptable to extreme conditions, including heavy metal contamination. Bacteria can evolve in the natural environment, generating resistant strains that can be studied to understand heavy-metal resistance mechanisms, but obtaining such adaptive strains usually takes a long time. In this study, the genome replication engineering assisted continuous evolution (GREACE) method was used to accelerate the evolutionary rate of the *Escherichia coli* genome to screen for *E. coli* mutants with high resistance to cadmium. As a result, a mutant (8mM-CRAA) with a minimum inhibitory concentration (MIC) of 8 mM cadmium was generated; this MIC value was approximately eightfold higher than that of the *E. coli* BL21(DE3) wild-type strain. Sequencing revealed 329 single nucleotide polymorphisms (SNPs) in the genome of the *E. coli* mutant 8mM-CRAA. These SNPs as well as RNA-Seq data on gene expression induced by cadmium were used to analyze the genes related to cadmium resistance. Overexpression, knockout and mutation of the *htpX* (which encodes an integral membrane heat shock protein) and *gor* (which encodes glutathione reductase) genes revealed that these two genes contribute positively to cadmium resistance in *E. coli*. Therefore, in addition to the previously identified cadmium resistance genes *zntA* and *capB*, many other genes are also involved in bacterial cadmium resistance. This study assists us in understanding the mechanism of microbial cadmium resistance and facilitating the application of heavy-metal remediation.

## Introduction

Cadmium (Cd) is a highly toxic, non-essential heavy metal element for all organisms (Abbas et al., 2017) that can accumulate in cells, where it disturbs the function of some enzymes and directly attacks nuclear DNA (Khan et al., 2015; Abbas et al., 2017). If humans consume cadmium-contaminated food, water or air, kidney, lung, liver, bone, and reproductive system diseases can occur. Cadmium is now designated a human carcinogen according to the International Agency for Research on Cancer (Muir and Weiland, 1995). With the rapid development of industry, heavy-metal pollution poses many risks to human health. However, some microbes survive especially well in environments that are extremely polluted with cadmium, as they have evolved various metal resistance mechanisms (Huang et al., 2014).

According to previous studies, bacterial strains resistant to cadmium have been isolated from heavy-metal-contaminated areas. Three metal resistance mechanisms in bacteria have been revealed: metal ions can be prevented from flowing into cells by the presence of polysaccharides, proteins and fats on their surfaces (Deb et al., 2013); heavy metals can be expelled through efflux pumps (Maynaud et al., 2014); and metal ions can be chelated by intracellular metallothionein (MT) (Sekhar et al., 2011; Shamim et al., 2014; Seifipour et al., 2016; Niederwanger et al., 2017).

*Escherichia coli*, a model organism, is often used in studies investigating microbial resistance to cadmium ions. The gene *zntA* in the genome of *E. coli* was shown to encode Pb^2+^-, Zn^2+^-, and Cd^2+^-transporting ATPases that confer cadmium tolerance to *E. coli* (Binet and Poole, 2000; Vidhyaparkavi et al., 2017); the *capB* gene that we identified in a previous study also enhances cadmium resistance in *E. coli* by binding to cadmium ions (Qin et al., 2017). In addition, some plasmids with cadmium resistance genes, such as pMOL30, which encodes the CzcABC efflux system, and pMOL28, which encodes the cnr system (Legatzki et al., 2003; Monchy et al., 2007), have been described. However, aside from these efflux pump and cadmium binding genes, whether other genes related to cadmium resistance exist in the genome of *E. coli* is unknown; furthermore, whether *E. coli* can evolve to a state of high cadmium resistance in the laboratory has not been determined.

In this study, we stimulated evolution in *E. coli* to achieve high-level cadmium resistance in the laboratory. The mutant plasmid PQ (which encodes a DNA polymerase III with reduced proofreading activity due to a modified 𝜀 subunit [*dnaQ*)] was used to accelerate genome evolution under heavy-metal pressure. Eventually, a mutant (8mM-CRAA) with a minimum inhibitory concentration (MIC) of 8 mM was generated; this MIC value was approximately eightfold higher than that of the *E. coli* BL21(DE3) wild-type strain (Luan et al., 2013, 2015). Single nucleotide polymorphisms (SNPs) in the mutants and RNA-Seq data on gene expression induced by cadmium were used to analyze genes related to cadmium resistance. Through an overexpression experiment, we identified two genes, *gor* [encoding glutathione oxidoreductase (Gor)] and *htpX* (an integral membrane heat shock protein), that contributed to the high cadmium resistance of BL21(DE3). To the best of our knowledge, no studies have reported that the *htpX* and *gor* genes can improve cadmium resistance in *E. coli*. A series of overexpression, knockout, complementation and site-specific mutagenesis experiments were performed, revealing that the two genes (*htpX* and *gor*) and specific residues in them (V16 in HptX and G249 in Gor) are important for conferring cadmium resistance to *E. coli*. Therefore, in addition to the previously known exporter protein ZntA and cadmium binding protein CapB, the proteins Gor and HtpX are involved in conferring cadmium resistance to BL21(DE3); these latter two proteins represent previously unknown cadmium resistance mechanisms.

## Results

### Improving the Cadmium Resistance of *E. coli* BL21(DE3) by Genome Replication Engineering-Assisted Continuous Evolution (GREACE)

**FIGURE 1:**
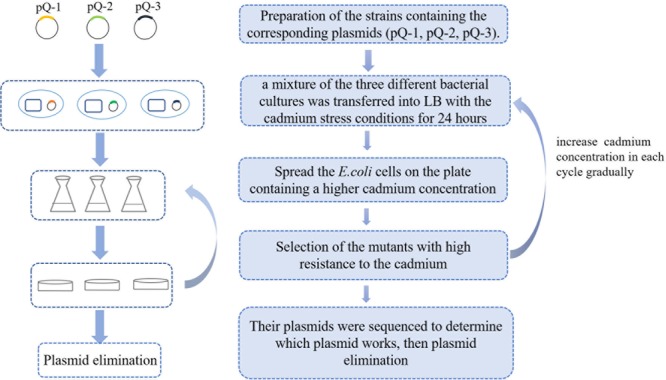
Flowchart of the genome replication engineering-assisted continuous evolution (GREACE) method. To improve cadmium resistance, pQ-1, pQ-2, and pQ-3 were transformed into host cells [BL21(DE3)] and cultivated under conditions with gradually increasing selective strength. Evolved strains with higher cadmium resistance were selected, and the plasmids were sequenced for confirmation. Genetically modified pQ mutants were eliminated from the evolved strains to stabilize the obtained genotypes and phenotypes.

To obtain a highly cadmium-resistant strain, we used the GREACE method to selectively grow wild-type *E. coli* BL21(DE3) harboring different mutant plasmids (pQ-1, pQ-2, or pQ-3); the details are shown in Figure 1. First, we cultured the negative control group (harboring the pUC19 vector) and the experimental group (harboring the mutant plasmid pQ-1) under 0.8 mM cadmium pressure [MIC value of *E. coli* BL21(DE3)] for 24 h. Next, we spread the cultures on Luria Bertani (LB) agar plates containing 2 mM Cd^2+^, 3 mM Cd^2+^, or 4 mM Cd^2+^. As shown in Supplementary Figure S1, the negative control strains were unable to grow on the plates containing 2 mM Cd^2+^, 3 mM Cd^2+^, or 4 mM Cd^2+^. However, some mutants containing the pQ-1 plasmid grew on these plates (Supplementary Figure S1). This result indicated that the pQ-1 plasmid mutator accelerated genome evolution in *E. coli*. Therefore, mutants with enhanced cadmium resistance could be obtained by screening on the plates.

**FIGURE 2:**
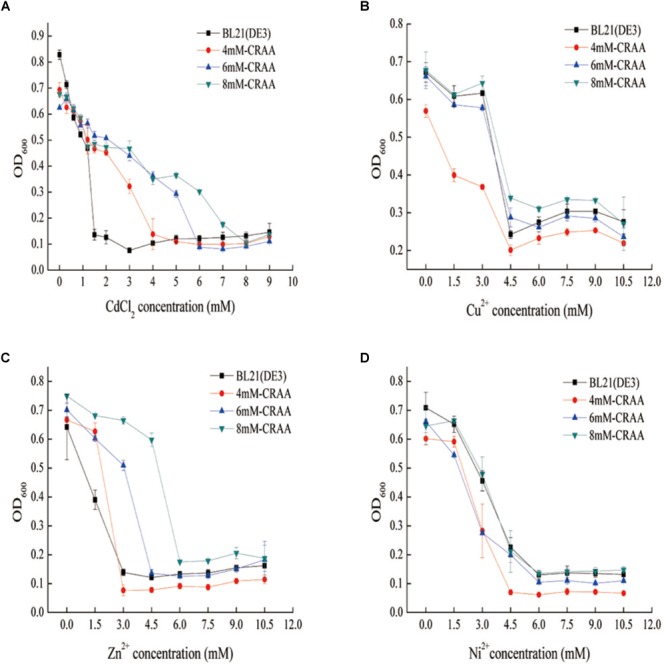
Determination of different heavy-metal resistance mechanisms in the mutant strains. **(A)** Resistance of mutant strains to Cd^2+^. **(B)** Resistance of mutant strains to Cu^2+^ (CuCl_2_). **(C)** Resistance of mutant strains to Zn^2+^ (ZnSO_4_). **(D)** Resistance of mutant strains to Ni^2+^ (NiSO_4_).

**FIGURE 3:**
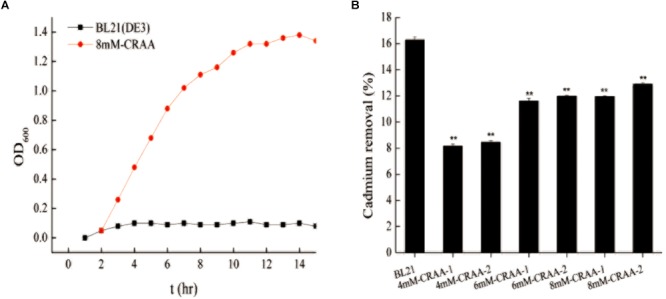
Growth curve of 8mM-CRAA in 1.2 mM Cd^2+^ and the cadmium removal capacity of different strains. **(A)** Growth curve of 8mM-CRAA in 1.2 mM Cd^2+^; BL21(DE3) was used as a negative control. **(B)** Cadmium removal capacity of different strains, with BL21 and pUC19-GFP as controls; ^∗∗^*p* < 0.01.

After iterative liquid and plate cultivation steps under gradually increasing cadmium concentrations, we obtained different cadmium-resistant strains (8mM-CRAA, 6mM-CRAA, and 4mM-CRAA). It took about 15, 20, and 25 rounds of domestication to generate mutant strains with cadmium resistance of 4, 6, and 8 mM, respectively. However, with the increase of cadmium pressure, the time of domestication needs to be increased accordingly. As shown in Figure 2A, the 8mM-CRAA, 6mM-CRAA, and 4mM-CRAA strains could grow in the presence of cadmium at concentrations up to 8, 6, and 4 mM cadmium, respectively, while the growth of wild-type BL21(DE3) was inhibited in the presence of 1 mM cadmium. The cadmium MIC value of strain 8mM-CRAA was eight times higher than that of wild-type *E. coli* BL21(DE3). We sequenced the plasmids in the cadmium-resistant strains (8mM-CRAA, 6mM-CRAA and 4mM-CRAA) and found that all of them were the strong mutator pQ-1 plasmid. To determine whether the mutant strain 8mM-CRAA indeed had a growth advantage under cadmium stress, we measured the growth curves of 8mM-CRAA and wild-type BL21(DE3) in medium with a cadmium concentration of 1.2 and 0 mM. As shown in Figure 3A, the growth of 8mM-CRAA was not affected by cadmium stress, while wild-type BL21(DE3) could not grow on such a plate. And interestingly, wild-type BL21(DE3) grew better than 8mM-CRAA in the absence of cadmium (Supplementary Figure S2). In addition, the cadmium adsorption capacities of different mutant strains were measured. As shown in Figure 3B, although cadmium resistance increased in the mutants, their cadmium adsorption capacity decreased. Therefore, highly cadmium-resistant strains may be capable of preventing more Cd^2+^ from entering into the cell or effluxing more Cd^2+^ than the wild-type strain.

To further explore whether the mutant strains had improved resistance to other heavy metals (Zn^2+^, Cu^2+^, and Ni^2+^), we measured the MIC values of the three strains. The results showed that Zn^2+^ resistance was also increased in the mutants (Figure 2B–D), likely because cadmium and zinc have similar physical and chemical properties and are located at the same group (IIB) of the periodic table. However, the MIC values of the mutants for the other two heavy metals (Cu^2+^, and Ni^2+^) were similar to those of wild-type *E. coli*, indicating that the evolutionary method used to improve the strains was directed. These data indicated that we successfully obtained strains with improved cadmium resistance within 2 months using the GREACE method, which is faster than the traditional domestication process.

### Genome Analysis of the 8mM-CRAA, 6mM-CRAA, and 4mM-CRAA Mutant Strains

To elucidate the mechanism of cadmium resistance in the mutant strains, we re-sequenced the genomes of the mutants (8mM-CRAA, 6mM-CRAA, and 4mM-CRAA). The SNP results revealed that the higher the resistances of the mutant strains, the more mutations were present in the genome (Supplementary Table S4). According to the SNP mutation results for the three strains in our study and two previous studies (Wang and Crowley, 2005; Helbig et al., 2008b) investigating the genome-wide analysis of temporal gene expression profiles in *E. coli* after exposure to different concentrations of cadmium, we screened ten genes that may be associated with cadmium resistance. The criteria for screening candidate genes were that all ten genes had mutations in strain 8mM-CRAA, and their mRNA expression levels were all upregulated at least 1.5 times by cadmium (Supplementary Table S1).

### Overexpression of the *gor* and *htpX* Genes Can Improve Cadmium Resistance in BL21(DE3)

**FIGURE 4:**
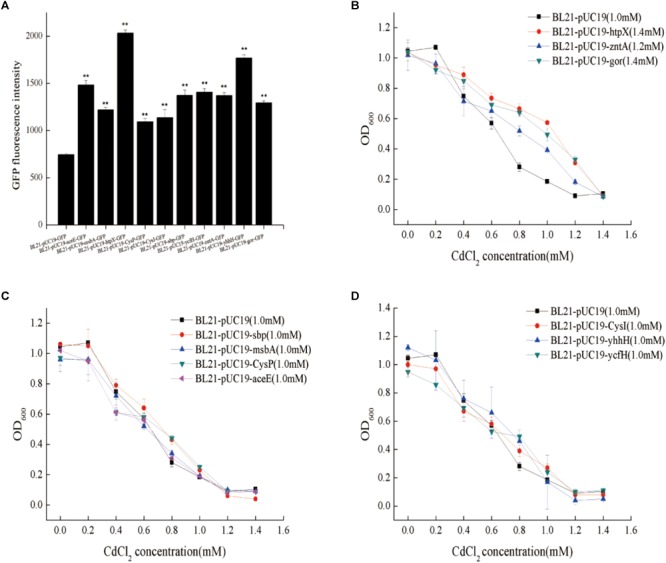
Fluorescence intensity and cadmium resistance capacity of gene-overexpressing strains. **(A)** BL21-pUC19-aceE-GFP and BL21-pUC19-sbp-GFP represent the overexpression of *aceE* and *sbp*, respectively, with BL21-pUC19-GFP as a negative control. **(B–D)** BL21-pUC19 was used as a control; ^∗∗^*p* < 0.01. 1.0 and 1.2 mM represent the cadmium MIC values.

To identify cadmium resistance gene(s), the 10 candidate genes (wild type) were separately overexpressed in BL21(DE3) to investigate whether they could increase the cadmium resistance of the strain. The selected genes were expressed as fusions with green fluorescent protein (GFP) to conveniently detect their expression by measuring fluorescence. As shown in Figure 4A, the ten genes were successfully overexpressed. However, overexpression of *gor* (glutathione oxidoreductase) and *htpX* (heat shock protein and integral membrane protein) had the most obvious influence on the cadmium resistance of BL21(DE3), achieving resistance levels even higher than that of *zntA*, which encodes the cadmium efflux gene (Figure 4B–D). Therefore, in the next step, we mainly focused on the genes *gor* and *htpX*.

### The *gor* and *htpX* Genes Are Important for Cadmium Resistance in BL21(DE3)

**FIGURE 5:**
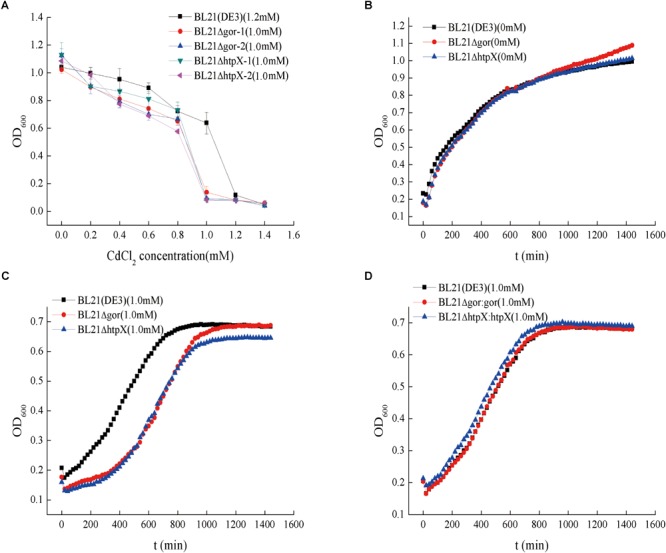
Cadmium resistance capacities and growth curves of knockout strains and plasmid remedial strains. The symbol “Δ” represents gene knockout. **(A)** Cadmium resistance capacities of the knockout strains, with BL21 as a control; BL21Δgor-1 and BL21Δgor-2 represent *gor* knockout strains. The 0.8 and 1.0 mM represent the cadmium MIC values. **(B)** Growth curves of the knockout strains without cadmium pressure. **(C)** Growth curves of the knockout strains with 1.0 mM cadmium ions. **(D)** Growth curves of the plasmid remedial strains with 1.0 mM cadmium ions. For the cadmium MIC assay **(A)**, 10-μL aliquots of overnight cultures of highly cadmium-resistant strains were transferred into 96-well plates (each well contained 1,000 μL of LB medium); for the growth assay **(B–D)**, 6-μL aliquots of overnight cultures were diluted into 100-well plates containing 350 μL of LB medium. The OD_600_ of the cell was measured with the instrument Bioscreen C every 20 min. The growth curve was the average value of 10 replicates.

To determine whether *gor* and *htpX* contribute to the cadmium resistance of BL21(DE3), we knocked out *gor* and *htpX* using CRISPR-Cas9 technology and named the resulting strains BL21ΔhtpX and BL21Δgor, respectively. The strains were verified by polymerase chain reaction (PCR) using specific primers (Supplementary Figure S3) to ensure that the two genes were indeed knocked out. In the absence of cadmium, the growth curves of the BL21ΔhtpX, BL21Δgor and wild-type strains were similar (Figure 5B). Subsequently, we measured their MIC values for Cd^2+^ (Figure 5A) as well as their growth curves with different cadmium concentrations (0 and 1.0 mM Cd^2+^). As shown in Figure 5A, the MIC values of the BL21ΔhtpX and BL21Δgor strains were about 0.2 mM lower than that of the wild-type strain. In addition, at a concentration of 1.0 mM Cd^2+^, the growth lag phases of BL21Δgor and BL21ΔhtpX were obviously prolonged (Figure 5C).

To further confirm these results, we transformed plasmids containing *htpX* and *gor* into BL21ΔhtpX and BL21Δgor to generate BL21ΔhtpX:htpX and BL21Δgor:gor, respectively. The cadmium resistance of the strains was determined. As shown in Figure 5D, the genes *htpX* and *gor* allowed BL21ΔhtpX and BL21Δgor, respectively, to recover their cadmium resistance capacity, similar to that of the wild-type BL21(DE3) strain. Overall, these results confirmed that the *gor* and *htpX* genes are associated with cadmium resistance in BL21(DE3).

### The V16 Residue of the HtpX Protein Plays an Important Role in Its Cadmium Resistance Function

**FIGURE 6:**
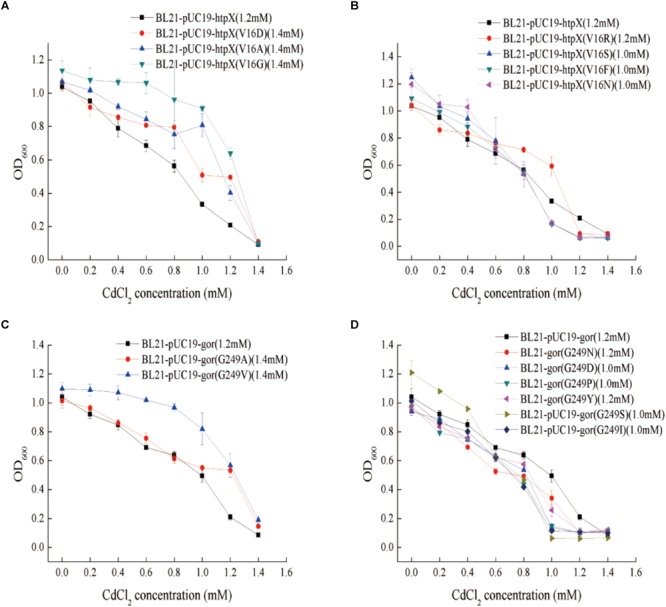
Determination of the cadmium resistance of different mutant strains. **(A,B)** Cadmium resistance of *htpX*-overexpressing strains, with BL21-pUC19 as a negative control; in BL21-pUC19-htpX (V16A), the *htpX* gene was overexpressed, and the V16 residue of HtpX was mutated from Val (V) to Ala (A). **(C,D)** Cadmium resistance of *gor*-overexpressing strains; in BL21-pUC19-gor (G249A), the *gor* gene was overexpressed, and the G249 residue of Gor was mutated from G (Gly) to Ala (A). 1.0 and 1.2 mM represent the cadmium MIC values.

According to the genome resequencing results for the 8mM-CRAA, 6mM-CRAA, and 4mM-CRAA strains, the V16 residue of the HtpX protein was mutated from Val (V) to Ala (A). Therefore, this mutation may have an effect on HtpX function. We then mutated this residue (V16) into some representative amino acids, including Arg (R), Asp (D), Gly (G), Ser (S), Phe (F), and Asn (N). As shown in Figure 6A,B, the cadmium resistance of these mutants was different. When V16 was mutated to A, D, or G, the MIC value increased by about 0.2 mM (Figure 6A). However, when the residue was mutated to R, S, F or N, the MIC value decreased by about 0.1–0.2 mM (Figure 6B). These results indicated that the residue V16 was important for HtpX to perform its cadmium resistance function.

In addition, HtpX plays an important role in the quality control of integral membrane proteins (Sakoh et al., 2005). Thus, we co-expressed *htpX* and *zntA* (a Zn^2+^, Cd^2+^ efflux transporter) to explore whether wild-type and mutant HtpX could intensify the function of ZntA and increase the cadmium resistance of the strain. However, co-expression did not improve the cadmium resistance capacity of the strain. Therefore, the mechanism of HtpX in cadmium resistance is not directly related to ZntA. The exact mechanism of HtpX remains to be studied.

### The G249 Residue of the Gor Protein Is Important for Its Cadmium Resistance Function

According to the genome resequencing results for the 8mM-CRAA, 6mM-CRAA, and 4mM-CRAA strains, the G249 residue of the Gor protein was mutated from G to A. To study the cadmium resistance mechanism of the G249 residue in Gor, the amino acid G249 was mutated to some representative amino acids, such as N, D, Pro (P), Tyr (Y), V, and S. As shown in Figure 6C,D, the cadmium resistance of the recombinants was different when G249 was replaced with other residues. When G249 was mutated to A or V, the MIC value of the recombinants increased by about 0.2 mM (Figure 6C). However, the MIC value decreased when the residue was mutated to D, Y, S, I, N or P (Figure 6D). Consequently, this residue is significant for the cadmium resistance function of Gor.

### The Gor Protein Influences the Glutathione (GSH) Content of Bacterial Cells

**FIGURE 7:**
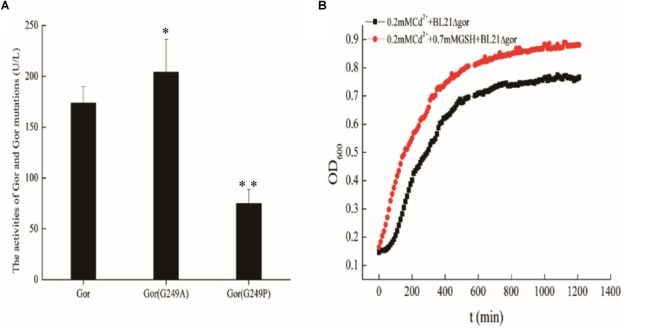
The effects of Gor mutations and growth curves of BL21Δgor with cadmium and GSH. **(A)** The effects of Gor mutations. A wild-type Gor strain was used as a control; ^∗^*p* < 0.05; ^∗∗^*p* < 0.01. **(B)** Growth curves of BL21Δgor with cadmium and GSH. Δ Represents gene deletion.

Previous studies have shown that cadmium stress affects the anabolism of GSH (Helbig et al., 2008a; Kim et al., 2018; Maity et al., 2018). Overexpression of the Gor protein may lead to an increase in GSH because it is involved in the GSH anabolic pathway. Consequently, we constructed the recombinant strains BL21-pUC19-gor, BL21-pUC19-gor (G249A), and BL21-pUC19-gor (G249P) to determine the activity of Gor, Gor (G249A) and Gor (G249P) by using a Glutathione Reductase activity assay kit. We also performed SDS–PAGE to ensure that the Gor proteins were successfully expressed (Supplementary Figure S4). In addition, since GOR expression is very low under its own promoter, we constructed *gor* and the two mutant genes on the pET30 background to further confirm the normal expression of GOR and its mutants (Supplementary Figure S5). As shown in Figure 7A, the activity of Gor (G249A) increased, and the activity of Gor (G249P) decreased relative to that of wild-type Gor, indicating that these mutations affect the activity of Gor.

Experiments in the field of chemistry usually prove the effect of a chemical substance by adding it directly into culture, so to further explore the cadmium resistance mechanism of Gor, we added 0.7 mM GSH and 0.2 mM Cd^2+^ to LB to determine the growth curve of BL21Δgor. A control experiment was also performed in the absence of 0.2 mM Cd^2+^ (as shown in Supplementary Figure S6). We observed that the addition of GSH improved the growth rate of BL21Δgor only in the medium containing cadmium (Figure 7B). These results indicated that Gor influences GSH content in cells, thus affecting their cadmium tolerance.

## Discussion

This study showed that microbes with high resistance to cadmium can be evolved in the laboratory. The GREACE method can exclude the effects of horizontal gene transfer and is beneficial for studying the characteristics of bacteria themselves. As a result, a mutant (8mM-CRAA) with a cadmium MIC value of 8 mM was generated; this MIC value was approximately eightfold higher than that of the *E. coli* BL21(DE3) wild-type strain.

**FIGURE 8:**
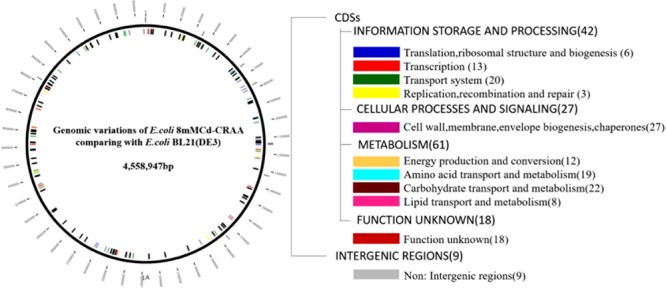
Map and categories of all genomic mutations in *Escherichia coli* 8mM-CRAA relative to its ancestor wild-type strain *E. coli* BL21(DE3). Mutations that occurred in coding sequences (CDSs) and intergenic regions are marked inside and outside of the left circle, respectively. The numbers in parentheses on the right are the total numbers of mutations in each category.

A fosmid library of the mutant (8mM-CRAA) was constructed and used to screen for genome fragments that could significantly enhance cadmium resistance. Unfortunately, although we conducted this experiment three times, we could not find a positive clone that increased the MIC value to 2 mM, indicating that many genes contribute to the cadmium resistance of the mutant strains, and these functional genes are located far away from each other in the genome. The resequenced genomes revealed many mutations in the mutant (8mM-CRAA) genome (Figure 8), including 20 transporter genes, 3 DNA replication genes, and many genes related to the metabolism of carbohydrates, amino acids and lipids. Microbes may activate whole metabolic systems to cope with the changes that occur under cadmium stress, and the genes may work together to endow the strain with high resistance to cadmium. In addition, the mutant genes are located far away from each other in the genome; thus, it was not possible for all of them to be included in a single clone in the constructed fosmid library. Thus, finding a better method to explore the multiple genes that contribute to cadmium resistance is required for future work.

In this study, we systematically identified two genes, *htpX* and *gor*, that are related to the cadmium resistance of *E. coli*. The *gor* gene encodes a glutathione reductase (GR), an enzyme that uses NAD(P) to convert oxidized glutathione (GSSG) to GSH (Miranda-Vizuete et al., 1996). Many studies have shown that cadmium can inhibit the activities of many antioxidant enzymes and reduce the level of the endogenous antioxidant GSH, decreasing its availability for clearing oxygen free radicals and causing mechanical damage (Thapa et al., 2016). However, the vast majority of studies on GR and GSH have been performed in plants, with few focused-on microorganisms. In this study, the overexpression of *gor* increased the cadmium resistance of BL21(DE3), and adding GSH improved the growth rate of BL21Δgor. Our results demonstrated that GSH biosynthesis also plays an important role in cadmium stress in *E. coli*.

However, the exact mechanism by which *htpX* exerted its effect was unclear. HptX is known to be a membrane-bound quality control factor and zinc-dependent endoprotease that belongs to the membrane-localized proteolytic system in *E. coli* (Shimohata et al., 2002). The expression of HtpX was upregulated in response to aminoglycoside exposure (Huang et al., 2018). In addition, some studies have confirmed that the activity of HtpX relies on the presence of Zn^2+^ (Kornitzer et al., 1991; Sakoh et al., 2005). Therefore, one explanation may be that Cd^2+^ is similar to Zn^2+^, and thus Cd^2+^ replaced Zn^2+^, binding to its metal binding site. However, the detailed mechanism needs to be investigated in further studies.

In conclusion, this study shows that bacteria can easily evolve high-level cadmium resistance in the laboratory and that many genes in the genome contribute to this special phenotype. In addition to the previously known proteins ZntA and CapB, the proteins Gor and HtpX also confer cadmium resistance in BL21(DE3).

## Materials and Methods

### Strains and Growth Conditions

*Escherichia coli* BL21(DE3) was used to screen for cadmium resistance strains. All strains were grown in LB medium (10 g L^-1^ tryptone, 5 g L^-1^ yeast extract, and 10 g L^-1^ NaCl) at 37°C with shaking at 200 rpm. All reagents used in this study were purchased from Sigma-Aldrich (St. Louis, MO, United States).

### Plasmid Instructions

The pQ-1 (pQ-*dnaQ*-BR1), pQ-2 (pQ-*dnaQ*-BR2), and pQ-3 (pQ-*dnaQ*-BR3) mutant plasmids were provided by Professor Yin Li, CAS Key Laboratory of Microbial Physiological and Metabolic Engineering, Institute of Microbiology, Chinese Academy of Sciences (Luan et al., 2013, 2015). The three plasmids all include the *dnaQ* promoter and *dnaQ* gene, but they have different mutations that improve the mutation rate of the genome and accelerate strain evolution. pQ-1 is a strong mutator that improves the natural mutation rate more than 2839-fold; pQ-2, a medium-intensity mutator, can improve the rate 317-fold; and pQ-3, a weak mutator, can increase the rate 30-fold. The pUC19 vector served as a negative control.

### The Domestication of Highly Resistant Cadmium Strains

*Escherichia coli* BL21(DE3) cells that were transformed with plasmids pQ-1, pQ-2, pQ-3, or pUC19 were transferred into fresh LB medium containing 100 μg/mL ampicillin and grown overnight. Then, a mixture of the three different bacterial cultures in broth (200 μL each of BL21-pQ-1, BL21-pQ-2, and BL21-pQ-3) was transferred into 30 mL of LB containing 100 μg/mL ampicillin and 0.8 mM Cd^2+^ [the critical concentration for growth of BL21(DE3)] and cultivated for 24 h. Meanwhile, an equal amount of BL21-pUC19 culture broth was transferred into the same medium as a negative control. Then, 200 μL of culture broth was spread on LB agar plates containing 100 μg/mL ampicillin and 2, 3 or 4 mM cadmium, followed by cultivation for 48 h. If single colonies grew on the 4 mM Cd^2+^ LB agar plate, we selected three colonies and inoculated them into 30 mL of liquid LB containing 1.0 mM cadmium for the second selection cycle; liquid and solid culturing were alternated. In each cycle, we increased the cadmium concentration in the liquid LB by 0.2 mM and the cadmium concentration of the solid LB by 1 mM until we obtained highly cadmium-resistant strains. This method is called GREACE (as shown in Figure 1).

### Plasmid Elimination

The plasmid in the final evolved strain was eliminated by serial transfer in LB without ampicillin but supplemented with corresponding cadmium selective pressure. An appropriate amount of culture was spread on agar plates with the same concentration of cadmium to obtain single colonies. Thirty single colonies were streaked on agar plates with ampicillin, and those with ampicillin sensitivity were regarded as plasmid-cured.

### Determination of the MIC of Cadmium

Minimum inhibitory concentration values were assessed by measuring the growth rates of different strains by estimating their optical density at 600 nm (OD_600_). In this study, the MIC values were defined as the cadmium concentration at which the 90% visible growth of organisms was inhibited. For the assay, 10 μL of overnight cultures of highly cadmium-resistant strains were transferred into 96-deep-well plates (each well contained 1,000 μL of LB medium) containing different concentrations of CdCl_2_ (0–10 mM) in triplicate. The plates were incubated at 37°C and agitated in an orbital shaker at 600 rpm (Inkubator 1000, Heidolph, Germany). After incubation at 37°C for 24 h, the OD_600_ was measured in an absorbance cuvette.

### Determination of Cadmium Removal

The wild-type BL21(DE3) and mutant strains 4mM-CRAA, 6mM-CRAA, and 8mM-CRAA were cultivated in LB broth for 12 h in a shaking incubator (200 rpm) before transferring them into 3 mL of LB supplemented with 0.1 mM Cd^2+^ for 24 h. Then, the cultures were transferred into 1.5-mL tubes and centrifuged at 10,000 *g* for 10 min. The supernatants were separated and diluted 100-fold using 0.6 M HCl, and the Cd^2+^ concentration was determined with an atomic absorption spectrophotometer (AAS). Based on the mean absorbance, the concentration of Cd^2+^ was determined by comparison with the mean absorbance of the standard Cd^2+^. The experiments were performed in triplicate.

### Determination of Growth Curves

To evaluate the tolerance of the GREACE-generated cells to cadmium, growth of the final isolated mutant strains was analyzed with serial concentrations of cadmium and compared with that of wild-type BL21(DE3). For the growth assay, *E. coli* cells were cultivated overnight at 37°C in LB medium, and then 6-μL overnight cultures were diluted into 100-well plates containing 350 μL of LB with different concentrations of CdCl_2_ in triplicate. We used an automatic growth curve analyzer to monitor the OD_600_ value at each time point in real time for 24 h.

### Genome-Wide Resequencing

Total genomic DNA was extracted from fresh bacterial broth cultures with a Bacterial Genome Rapid Extract Kit (TIANGEN, Beijing, China), according to the manufacturer’s protocol. DNA concentration and quality were checked by absorbance (A_260_/A_280_∼1.8) and gel electrophoresis (1%). Then, the genomes of the *E. coli* mutants with improved cadmium resistance were re-sequenced using the Illumina sequencing platform. The sequenced data of samples (8mM-CRAA, 6mM-CRAA, and 4mM-CRAA) could be downloaded from the China National GenBank (CNGB), which accession numbers are CNX0028499, CNX0028500, and CNX0028501, respectively. SNPs in the mutants and RNA-Seq data on gene expression induced by cadmium were used to analyze the genes related to cadmium resistance.

### Gene Overexpression in *E. coli*

Genes and their promoters predicted using Web Promoter Scan Services^1^ was amplified by PCR from the BL21(DE3) genome with the primers listed in Supplementary Table S2 and digested with *Kpn*I and *Xba*I. These fragments were inserted into *Kpn*I/*Xba*I double-digested pUC19-GFP, which contains the GFP reporter gene *gfp*, to create recombinant plasmids. Each plasmid was verified by DNA sequencing and transformed into BL21(DE3) to express the GFP-tagged proteins CysP, CysI, HtpX, Gor, AceE, ZntA, YcfH, MsbA, YhhH, and Sbp, separately. All hosts harboring the recombinant vectors were grown in LB at 37°C for 20 h. To confirm gene expression, we measured the fluorescence of the cultures using a fluorescence absorbance cuvette. Fluorescence in the wells was measured at an excitation/emission wavelength of 484/507 nm using a microplate reader (SpectraMax M2, Molecular Devices, United States).

### Site-Directed Mutagenesis

Site-directed mutagenesis of *htpX* and *gor* was performed using a two-step PCR method, as described previously. Primers containing mutation sites in the middle of their sequences are shown in Supplementary Table S3. The initial PCR was performed in a 50-μL volume using 2.5 U of Fast Pfu DNA polymerase, the provided Fast Pfu Buffer, each dNTP at 200 μM, 50 ng of the BL21(DE3) genome, and each primer at 1 μM. PCR amplifications were performed using the following program: 95°C for 5 min; 30 cycles of 95°C for 30 s, 58°C for 30 s, and 72°C for 40 s; and a final step at 72°C for 10 min. The DNA fragments were purified using a Universal DNA Purification Kit (TIANGEN, Beijing, China) and were eluted in water.

Next, 500 ng of the fragments produced in the first PCR were used as megaprimers, with 50 ng of pUC19-htpX or pUC19-gor as the template, 2.5 U of Fast Pfu polymerase, and 200 μM dNTPs in 50-μL reactions. The following amplification program was used: 95°C for 5 min, 30 cycles of 95°C for 30 s and 68°C for 5 min, and a final step at 72°C for 16 min. Subsequently, digestion with 10–20 U of *Dpn*I was performed at 37°C for 2 h. Recycling purification of the PCR products was performed using the Universal DNA Purification Kit. The PCR products were then transformed into *E. coli* BL21(DE3) competent cells.

### Using CRISPR-Cas9 Technology to Knock Out Genes

*Escherichia coli* BL21(DE3) knockout mutants were constructed by the CRISPR-Cas9 method, using the plasmids pCas (Addgene plasmid #62225) and pTargetF (Addgene plasmid #62226) as previously described (Jiang et al., 2015). To knock out the genes of interest, upstream and downstream fragments of each gene were amplified separately from BL21(DE3) genomic DNA with the corresponding UF/UR and DF/DR primers (Supplementary Table S3), and a single guide RNA (sgRNA) containing a targeting N20 sequence of the locus of interest was amplified from pTF using the corresponding SPF primers with the common reverse primer sgRNA-R. These three fragments were combined via an overlap PCR with the SPF and DR primers, digested with *Spe*I/*Sal*I and inserted into *Spe*I/*Sal*I-digested pTF to construct the knockout plasmids (Supplementary Table S3). These plasmids were then transformed into BL21(DE3) harboring pCas, and the deletion strains were screened as previously described. Gene knockouts were verified by colony PCR with primers that were positioned ∼800 bp upstream and downstream of the target genes.

### Plasmid Complementation

The recombinant plasmids pUC19-htpX and pUC19-gor were constructed to express the genes *hptx* and *gor*. These plasmids were transformed into BL21(DE3) ΔhtpX and BL21(DE3)Δgor, respectively. To verify that the recombinant plasmids were successfully transformed, we performed PCR using the pUC19 universal primers M13F/M13R.

### Determination of Gor Activity

Overnight cultures of the recombinant BL21-pUC19-gor and BL21-pUC19-gor(G249P) strains were transferred into 50 mL of LB medium supplemented with ampicillin at a final concentration of 100 μg/mL. The recombinant strains BL21-pUC19-gor and BL21-pUC19-gor(G249P) contained the expression plasmids for wild-type Gor and the mutated versions, respectively. After incubation at 37°C for 1.5–2 h, the cultures were adjusted to an OD_600_ value of 0.8–1.0. Target proteins were released from the cells by sonication. The lysate supernatants were then collected by centrifugation at 12,000 rpm for 10 min and used to measure the activity of Gor with a Glutathione Reductase Activity Assay Kit, according to the manufacturer’s instructions (Nanjing Jian Cheng, China). The following specific formula was used to calculate Gor enzyme activity:

$$\begin{array}{c} Gor activity\left(U/L\right)=\left(A1-A2\right)/\left[6.22^{*}cuvette diameter\left(1 cm\right)\right]/reaction time{\left(2 min\right)}^{*}[1,000\;\left(\mathrm{mL}\right)/sampling quantity\left(0.05 mL\right)] \end{array}$$

### Statistics Analysis

The statistics analysis in this study was calculated with the software R and its “stats” library. The differences between the two samples were calculated with the method of *t*-test. The *p*-value less than 0.05 represents statistically significant (^∗^) and *p*-value less than 0.01 represents very significant (^∗∗^).

## Author Contributions

JT and NW designed the study. WQ, JZ, XY, XC, and XL performed the experiments. WQ and JZ analyzed the data. WQ, JZ, NW, and JT wrote the manuscript. All authors participated in the discussion of the research and approved the final manuscript.

## Conflict of Interest Statement

The authors declare that the research was conducted in the absence of any commercial or financial relationships that could be construed as a potential conflict of interest.

## Footnotes

1 http://www-bimas.cit.nih.gov/molbio/proscan
