# Supplementary material for: Improving Cadmium Resistance in Escherichia coli Through Continuous Genome Evolution
Source: Front Microbiol. 2019 Feb 20;10:278. doi: 10.3389/fmicb.2019.00278 (PMC6391850; doi:10.3389/fmicb.2019.00278)
Supplement: Supplementary file 1 [file Data_Sheet_1.PDF]

Supplementary materials

TABLE S1 Descriptions of the 10 candidate genes.

Gene name	WT ± Cd (25 μM) transcriptional level (fold change)	WT ± Cd (100 μM) transcriptional level (fold change)	SNP variant (numbers)	Description
ycfH	2.16	1.05	6	ORF, hypothetical protein
zntA	7.26	9.51	1	Zinc-transporting ATPase
cysI	3.28	8.57	1	Sulfite reductase, α subunit
msbA	1.66	11.99	1	ATP-binding transport protein; multicopy suppressor of htrB
gor	1.52	5.29	2	Glutathione oxidoreductase
aceE	1.51	4.06	1	Pyruvate dehydrogenase
htpX	1.57	1.73	1	Heat shock-protein, integral membrane protein
yhhH	127.79	0.60	1	ORF, hypothetical protein
sbp	2.32	46.52	1	Periplasmic sulfate-binding protein
cysP	4.04	22.17	1	Thiosulfate binding protein

TABLE S2 The primers of ten candidate genes relating to cadmium resistance.

Primer name	Sequence (5'-3')
GFP-F	AACTGCAGATGGTGTCCAAAGGTGAAG
GFP-R	CCCAAGCTTTTATTTATACAATTCATCCATACCTAAC
aceE-F	GGGGTACCTCAAGTTGTTAAAATGTGCAC
aceE-R	GCTCTAGACGCCAGACGCGGGTTAACTTTATCT
CysI-F	GGGGTACCATCACCGCACTTAACAG
CysI-R	GCTCTAGAATCCCACAAATCGCG
CysP-up	GGGGTACCAATGGCGGAAAGCATTG
CysP-down	GCTCTAGATTACGCCCCGCCGCTA
gor-up	GGGGTACCAGCAAATTAAGCGCATGATC
gor-down	GCTCTAGAACGCATTGTCACGAACT
htpX-up	GGGGTACCACGGGTAATGAAGAAACG
htpX-down	GCTCTAGACTTCAGGTATTCACCCGT
msbA-up	GGGGTACCTTCTGAGCCATGAACATCTG
msbA-down	GCTCTAGATTGGCCAAACTGCATTTTG
sbp-up	GGGGTACCTTAGCCTGAAACCGATGA
sbp-down	GCTCTAGAGCGTTTGCTGATCTGAT
ycfH-up	GGGGTACCTGGATCGTATTGAGCAAGAG
ycfH-down	GCTCTAGAACGGATGGATTGAAGGCGGGA
yhhH-up	GGGGTACCTGGTCTTTGCGGCACAC
yhhH-down	GCTCTAGATTTTCCGTGGCCAAAATTC
ZntA-up	GGGGTACCAGGTGAACTGTGGTTCTTC
ZntA-down	GCTCTAGATCTCCTGCGCAACAATC

TABLE S3 Genes knockout and mutant primers of htpX and gor.

Primer name	Sequence (5'-3')
SgRNA-R	TCAAAAAAAGCACCGACTCGG
SgRNA-htpX-F	TCCTAGGTATAATACTAGTtggccgtaatggtcgttttcGTTTTAGAGCTAGAAATAGC
SgRNA-gor-F	TCCTAGGTATAATACTAGTatcaaccgcgcggctatgtaGTTTTAGAGCTAGAAATAGC
L-htpX-F	CCGAGTCGGTGCTTTTTTTGAGAAACAGGTTTACTTCTGACACC
L-htpX-R	CCAGACTTGAAAATAGTCGCGTA ATACTAAAAAGCGCGTCCTG
R-htpX-F	TACGCGACTATTTTCAAGTCTGG
R-htpX-R	TCCCCGCGGTGAAATCAGGCGATTTAACG
L-gor-F	CCGAGTCGGTGCTTTTTTTGAAACACTTCAACCGTAGCG
L-gor-R	CACTACTCTTAGCCCTTTAACATTGACAAAGTGTCCTTATCGTT
R-gor-F	AATGTTAAAGGGCTAAGAGTAGTG
R-gor-R	TCCCCGCGGTTAGCCAGTGGCTAACATTG
htpX(V16S)-F	ACCTGGCCGTAATGCACGTTTTCGGGCTGG
htpX(V16F)-F	ACCTGGCCGTAATAACCGTTTTCGGGCTGG
htpX(V16N)-F	ACCTGGCCGTAATAATCGTTTTCGGGCTGG
htpX(G16D)-F	ACCTGGCCGTAATGGACGTTTTCGGGCTGG
htpX(G16R)-F	ACCTGGCCGTAATGCGCGTTTTCGGGCTGG
htpX(G16Y)-F	ACCTGGCCGTAATGTACGTTTTCGGGCTGG
htpX-R	ACGATGACGCGAGAACCAC
Gor(G249D)-F	CTGGAGCTGGAAGATGATCGCAGTGAAACGGTGG
Gor(G249Y)-F	CTGGAGCTGGAAGATTATCGCAGTGAAACGGTGG
Gor(G249N)-F	CTGGAGCTGGAAGATAATCGCAGTGAAACGGTGG
Gor(G249P)-F	CTGGAGCTGGAAGATCCTCGCAGTGAAACGGTGG
Gor(G249R)-F	CTGGAGCTGGAAGATCGACGCAGTGAAACGGTGG
Gor(G249A)-F	CTGGAGCTGGAAGATGCACGCAGTGAAACGGTGG
Gor(G249S)-F	CTGGAGCTGGAAGATTCGCGCAGTGAAACGGTGG
Gor(G249I)-F	CTGGAGCTGGAAGATATCCGCAGTGAAACGGTGG
Gor-R	GTCTTTTTTGGTTGCCCCCATCTTC

TABLE S4 The SNP Mutant number of different mutant genomes.

	4mMCd-CRAA	6mMCd-CRAA	8mMCd-CRAA
SNP mutant numbers	286	307	329

FIG. S1

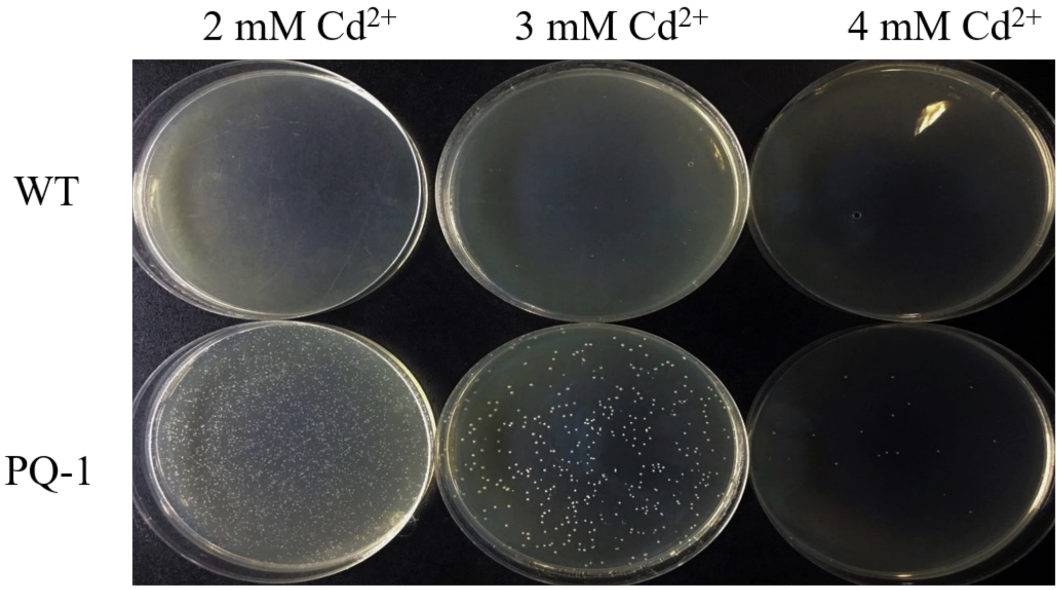

FIG. S1 Validation the role of PQ-1 plasmid. WT represents wild-type strains containing pUC19 plasmid; PQ-1 represents mutant strains containing pQ-1 plasmid; 2 mM Cd^{2+}, 3 mM Cd^{2+}, and 4 mM Cd^{2+} represent the different concentrations of cadmium in solid LB media.

FIG. S2

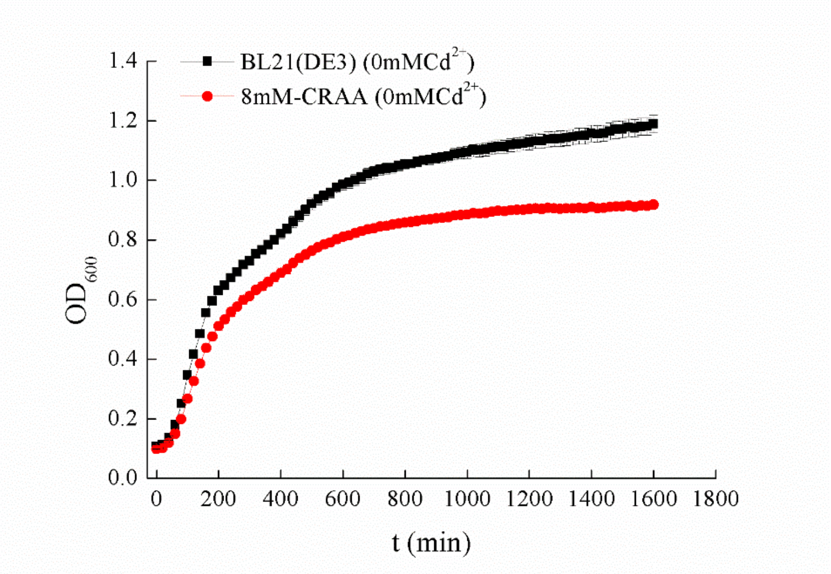

FIG. S2 Growth curves of wild-type BL21(DE3) and 8mM-CRAA in the absence of cadmium.

FIG. S3

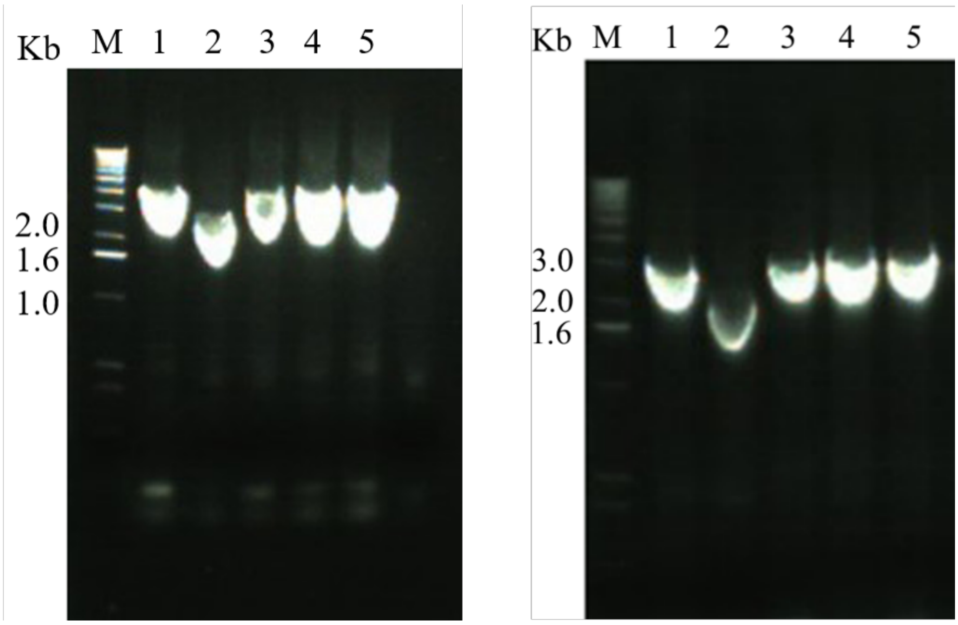

FIG. S3 PCR for isolation of knockout strains. Left: gene *gor* was successfully knocked out in lane 2; Right: gene *htpX* was successfully knocked out in lane 2.

FIG. S4

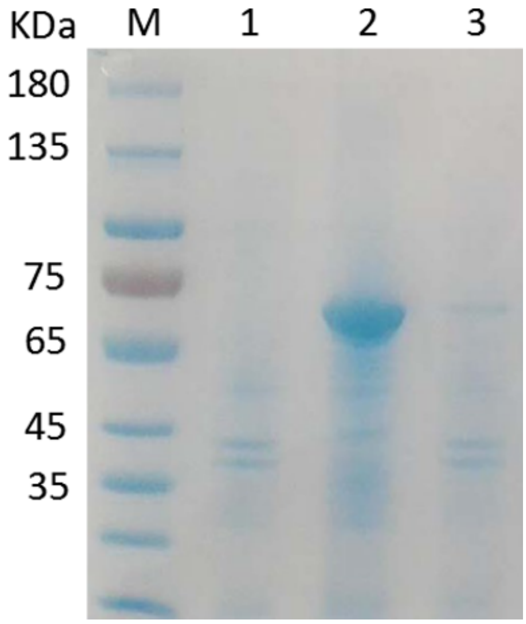

FIG. S4. The SDS-PAGE of Gor protein. M: Marker, lanes1, 2 and 3, negative control BL21-PUC19, BL21-pET30-gor and BL21-pUC19-gor, respectively.

FIG. S5

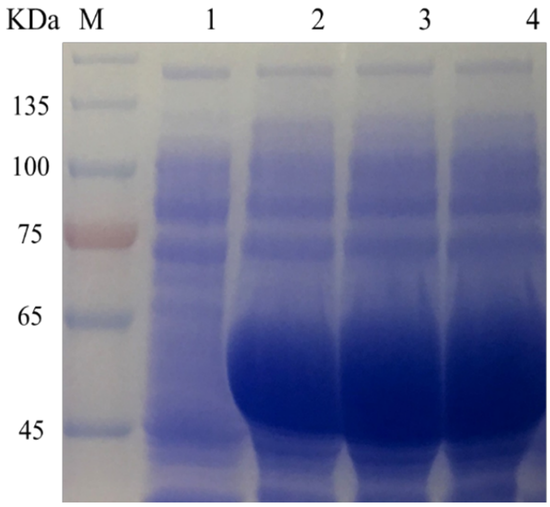

FIG. S5 The SDS-PAGE of Gor protein. M: Marker, lanes1, 2 and 3, negative control BL21-pET30, BL21-pET30-Gor, BL21-pET30-Gor(G249A) and BL21-pET30-Gor(G249P), respectively.

FIG. S6

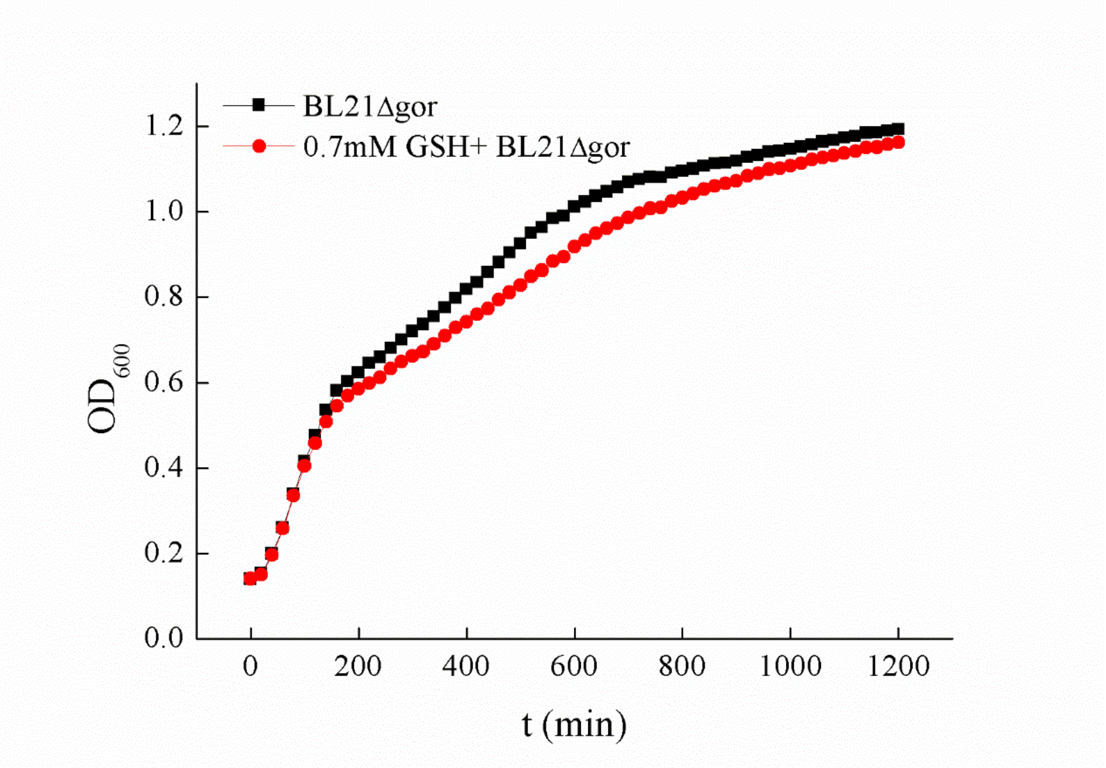

FIG. S6 The growth curves of BL21△gor with 0.7 mM GSH in the absence of cadmium.
